# Personality variability during development predicts alcohol use before and during the COVID-19 pandemic

**DOI:** 10.1038/s41598-026-63441-y

**Published:** 2026-08-14

**Authors:** Rabia Zohair, Karina Janson, Sebastian Siehl, Arun L. W. Bokde, Sylvane Desrivières, Hugh Garavan, Penny Gowland, Antoine Grigis, Andreas Heinz, Jean-Luc Martinot, Marie-Laure Paillère Martinot, Eric Artiges, Dimitri Papadopoulos Orfanos, Tomáš Paus, Luise Poustka, Michael N. Smolka, Nathalie E. Holz, Nilakshi Vaidya, Henrik Walter, Robert Whelan, Gunter Schumann, Tobias Banaschewski, Herta Flor, Olaf Reis, Emanuel Schwarz, the IMAGEN Consortium, Tianye Jia, Gareth J. Barker, Arun L. W. Bokde, Rüdiger Brühl, Sarah Hohmann, Sabina Millenet, Juliane H. Fröhner, the CoviDrug Consortium, Stella Guldner, Maren Prignitz, Elisa Wandinger, Antonia Fröhlich, Ersoy Kocak, Christoph Borzikowsky, Frauke Nees

**Affiliations:** 1 Institute of Medical Psychology and Medical Sociology, University Medical Center Schleswig-Holstein, Kiel University, Kiel, Germany; 2 Department of Child and Adolescent Psychiatry and Psychotherapy, Medical Faculty Mannheim, Central Institute of Mental Health, Heidelberg University, Square J5, 68159 Mannheim, Germany; 3 Discipline of Psychiatry, School of Medicine and Trinity College Institute of Neuroscience, Trinity College Dublin, Dublin, Ireland; 4 Centre for Population Neuroscience and Precision Medicine (PONS), Institute of Psychiatry, Psychology & Neuroscience, SGDP Centre, King’s College London, London, UK; 5 Departments of Psychiatry and Psychology, University of Vermont, Burlington, VT 05405 USA; 6 Sir Peter Mansfield Imaging Centre School of Physics and Astronomy, University of Nottingham, University Park, Nottingham, UK; 7 Université Paris-Saclay, CEA, CNRS, BAOBAB, Gif-sur-Yvette, 91191 France; 8 Department of Psychiatry and Psychotherapy CCM, Charité – Universitätsmedizin Berlin, corporate member of Freie Universität Berlin, Humboldt-Universität zu Berlin, Berlin Institute of Health, Berlin, Germany; 9 Department of Child and Adolescent Psychiatry, INSERM U A10 Trajectoires développementales & psychiatrie, AP-HP. Sorbonne Université, Pitié-Salpêtrière Hospital, Institut National de la Santé et de la Recherce Médicale, University Paris-Saclay, Ecole Normale Supérieure Paris- Saclay, CNRS; Centre Borelli, Gif-sur-Yvette, Paris, France; 10 Gif-sur-Yvette; and Psychiatry Department, Institut National de la Santé et de la Recherche Médicale, INSERM U A10 Trajectoires développementales en psychiatrie, Université Paris-Saclay, Ecole Normale supérieure Paris- Saclay, CNRS, Centre Borelli, EPS Barthélémy Durand, Etampes, France; 11 Department of Psychiatry, Faculty of Medicine and Centre Hospitalier Universitaire Sainte- Justine, University of Montreal, Montreal, QC Canada; 12 Departments of Psychiatry and Psychology, University of Toronto, Toronto, ON Canada; 13 Department of Psychiatry and Neuroimaging Center, Technische Universität Dresden, Dresden, Germany; 14 German Center for Mental Health (DZPG), Partner Site Mannheim-Heidelberg-Ulm, Mannheim, Germany; 15 Centre for Population Neuroscience and Stratified Medicine (PONS), Department of Psychiatry and Neuroscience, Charité Universitätsmedizin Berlin, Berlin, Germany; 16 School of Psychology and Global Brain Health Institute, Trinity College Dublin, Dublin, Ireland; 17 Centre for Population Neuroscience and Stratified Medicine (PONS), Institute for Science and Technology of Brain-inspired Intelligence (ISTBI), Fudan Universität, Shanghai, China; 18 Institute of Cognitive and Clinical Neuroscience, Medical Faculty Mannheim, Central Institute of Mental Health, Heidelberg University, Square J5, Mannheim, Germany; 19 Department of Psychology, School of Social Sciences, University of Mannheim, 68131 Mannheim, Germany; 20 Department of Child and Adolescent Psychiatry, University Medical Centre Rostock, German Center for Child and Adolescent Health (DZKJ), Partner site Greifswald/Rostock, Rostock Germany, Gehlsheimer Str. 20, 18147 Rostock, Germany; 21 Department of Psychiatry and Psychotherapy, Medical Faculty Mannheim, Central Institute of Mental Health, Heidelberg University, Mannheim, German Germany; 22 Hector Institute for Artificial Intelligence in Psychiatry, Medical Faculty Mannheim, Central Institute of Mental Health, Heidelberg University, Mannheim, Germany; 23 Institute of Medical Psychology, LMU Medizin, Ludwig- Maximilians-Universität München, Goethestraße 31/I, 80336 München, Germany; 24 Department of Neuroimaging Institute of Psychiatry, Psychology & Neuroscience, King’s College London, London, UK; 25 Physikalisch-Technische Bundesanstalt (PTB), Braunschweig and Berlin, Germany

**Keywords:** Adolescents, Personality development, Alcohol abuse, COVID-19 pandemic, Negative life events, Diseases, Health care, Psychology, Psychology, Risk factors

## Abstract

Changes in personality during adolescence may shape future alcohol use, especially in times of societal crisis. The COVID-19 pandemic disrupted social norms and stress regulation, making it a natural experiment to examine how early-life personality trajectories predict later substance use. Using longitudinal data from the European IMAGEN cohort (N = 968), we investigated whether intra-individual personality changes during adolescence predicted alcohol use at three timepoints: pre-pandemic, immediately after COVID-19 onset (T1), and several months into the pandemic (T2). Generalized Additive Mixed Models (GAMMs) were used to model non-linear relationships between adolescent personality change (e.g., in anxiety sensitivity and openness) and alcohol use frequency across time. Participants were drawn from the IMAGEN cohort (N = 968; 54.4% female), assessed longitudinally from early adolescence (BL: M = 13.93, SD = 0.41) through middle (FU1: M = 16.04, SD = 0.60) and late adolescence (FU2: M = 18.41, SD = 0.65) to early adulthood (FU3: M = 22.00, SD = 0.66), with additional follow-ups during the COVID-19 pandemic (COVID-T1: M = 25.00, SD = 0.81; COVID-T2: M = 25.56, SD = 0.75). Assessments were conducted at approximately 2-year intervals during adolescence and at shorter intervals (several months) during the COVID-19 period. Analyses controlled for sex and country-level lockdown stringency. Intra-individual fluctuations in anxiety sensitivity significantly predicted increased alcohol use several months into the pandemic (T2; EDF = 1.86, p < 0.05), but not immediately after lockdown (T1). Pre-pandemic alcohol use was instead associated with changes in openness (EDF = 1.00, p < 0.05). Exploratory analyses suggested gender differences in anxiety-related drinking risk. Our findings suggest that personality dynamics in adolescence shape alcohol use responses during societal crises. Different traits appear to matter depending on the temporal and emotional context of the stressor. These findings have implications for personalized prevention efforts targeting stress-related substance use in youth.

**Supplementary Information:**

The online version contains supplementary material available at 10.1038/s41598-026-63441-y.

## Introduction

Alcohol use is a major contributor to global disease burden and early mortality, with wide-ranging consequences for physical health, cognitive functioning, and mental well-being^1–3^. Personality traits are among the most robust predictors of substance use across this period^4^. Both stable trait levels and within-person developmental changes have been linked to alcohol-use trajectories^5–12^. For example openness to experience has been linked to greater engagement in exploratory, novelty-seeking, and socially oriented behaviors, which may increase exposure to alcohol use opportunities during adolescence and young adulthood^13,14^. Individuals high in openness are more likely to seek new experiences and social stimulation, factors that have been associated with recreational and socially motivated drinking^15,16^. Consequently, fluctuations in openness during adolescence may reflect instability in exploratory and social behavioral tendencies that are relevant for alcohol use in normative, non-crisis contexts.

The Social Investment Principle and the Maturity Principle theories postulate that personality development is shaped by individuals’ increasing commitment to socially structured roles, such as education, work, and intimate relationships. As adolescents transition into adulthood, investment in these roles is associated with adaptive personality changes, including increases in conscientiousness and emotional stability^17,18^. Similarly, the Maturity Principle describes a general developmental trend toward greater psychological adjustment across adolescence and early adulthood, characterized by increases in socially desirable traits (e.g., conscientiousness, agreeableness) and decreases in traits associated with emotional instability (e.g., neuroticism). Importantly, these frameworks primarily describe normative developmental trajectories at the group level. However, individuals may differ in the extent to which they follow these patterns, and such deviations—reflected in greater intra-individual variability—may indicate less stable personality development and reduced capacity for adaptive self-regulation. Importantly, personality development is not only reflected in mean-level or rank-order changes, but also in the stability or variability of traits within individuals over time. Adolescents also show intra-individual variability, reflecting fluctuations around their typical levels of a trait over time. Such variability may signal instability in underlying regulatory systems, including emotional regulation and coping capacities, and has been associated with heightened vulnerability to internalizing symptoms and maladaptive behaviours^19–22^. In this context, variability may reflect affective dysregulation, defined as difficulties in maintaining stable and adaptive emotional responses over time. Individuals characterized by affective dysregulation often exhibit heightened emotional reactivity and reduced capacity to regulate negative affect, which has been linked to maladaptive coping strategies such as substance use^23,24^. While trait levels capture an individual’s average disposition, variability reflects the consistency of that disposition across time, and may therefore provide additional information about an individual’s capacity to respond adaptively to stress. However, the predictive value of variability — as opposed to trait levels — for later alcohol use remains underexplored, despite evidence that fluctuating affective or behavioural patterns may increase susceptibility to stress-related drinking.

Sex differences are also relevant when examining the relationship between personality and alcohol use. Prior research has shown that males and females differ in both drinking patterns and underlying motives, with females more likely to engage in coping-motivated drinking under conditions of stress, whereas males more often report social or enhancement motives (e.g^15^). In addition, traits such as anxiety sensitivity and emotional instability may be differentially expressed across sexes, potentially leading to sex-specific associations between personality variability and alcohol use.

One such global crisis was the COVID-19 pandemic. Beyond its health and socioeconomic impacts, the pandemic created widespread psychological stress, social isolation, and uncertainty, particularly among adolescents and young adults^25–28^. Alcohol use during this period showed heterogeneous patterns, including both increases and decreases, depending on context and subgroup^26,29–32^. In early adulthood, lockdowns often reduced social drinking opportunities, but several studies also reported pandemic-related increases in coping-motivated alcohol use, particularly among individuals experiencing heightened anxiety, loneliness, or stress^33–36^. These heterogeneous patterns suggest that individual vulnerability factors play a key role in shaping alcohol use responses under conditions of widespread stress.

Given that personality traits are thought to shape how individuals perceive and respond to stress, and that adolescence is a sensitive period for both personality development and substance use initiation, a critical question arises: Do intra-individual changes in personality during adolescence predict alcohol use in young adulthood—particularly during high-stress societal events like the COVID-19 pandemic?

Moreover, it is unclear whether the same personality factors that predict alcohol use pre-pandemic remain relevant during crisis periods, or whether different traits gain predictive power under conditions of collective stress. For instance, anxiety sensitivity - a trait reflecting fear of anxiety-related sensations - has been linked to coping-motivated drinking, particularly in women^37^. Openness to experience, in contrast, is often associated with exploratory social behaviors that may drive social drinking in low-stress environments. However, it remains unclear whether these pathways generalize to high-stress contexts such as a major public health crisis, which gains importance given the many challenges individuals had to cope with during this pandemic period^38^. Importantly, stress-inducing environments such as strict lockdowns may further amplify individual differences by increasing psychological distress while simultaneously limiting access to adaptive coping resources such as social support and structured daily activities^39,40^. Under such conditions, individuals with greater instability in personality traits may be more likely to engage in maladaptive coping behaviors, including alcohol use.

The present study addresses this gap using data from the IMAGEN cohort, a large-scale longitudinal study following adolescents from early adolescence into young adulthood^41^. We examined whether intra-individual variability^42^ in personality traits during adolescence predicts alcohol use at three timepoints: pre-pandemic early adulthood, immediately after the onset of COVID-19 (COVID-T1), and several months into the pandemic (COVID-T2). We operationalized variability as the statistical variance of repeated personality assessments across four adolescent waves, capturing the extent of individual fluctuations around typical trait levels, and thereby indexing the stability versus instability of personality development across adolescence. This approach is informed by developmental personality theories, such as the maturity and social investment principles, which suggest that personality typically becomes more stable and adaptive over time. From this perspective, greater intra-individual variability may reflect less consolidated self-regulatory processes and reduced stability in emotional and behavioral functioning.

At the same time, models of alcohol use emphasize the role of stress and coping, proposing that individuals are more likely to engage in alcohol use when experiencing negative affect or diminished regulatory capacity (e.g^15,43^). Integrating these perspectives, personality variability may represent a marker of vulnerability that becomes particularly relevant under conditions of stress, such as during the COVID-19 pandemic. In addition, environmental context may influence the extent to which personality-related vulnerabilities translate into behavior. During the COVID-19 pandemic, government-imposed restrictions varied substantially across countries, affecting levels of social isolation, stress, and access to coping resources. Stricter lockdown measures have been associated with increased psychological distress and changes in substance use patterns (e.g.,^39^,^31^). Therefore, country-level lockdown stringency was included as a contextual moderator to examine whether environmental constraints amplify or attenuate the association between personality variability and alcohol use.

We formulated three hypotheses.

1. Greater variability in anxiety sensitivity and impulsivity during adolescence would predict higher alcohol use during the pandemic—particularly at COVID-T2, when stress-related drinking was expected to increase—and more strongly in females.
2. Variability in openness would be more strongly associated with pre-pandemic alcohol use, reflecting its link to social and exploratory drinking motives.
3. Country-level lockdown stringency would moderate associations between personality variability and alcohol use, such that stricter restrictions would amplify trait-related risk.

By linking adolescent personality development to alcohol-use responses in the face of a major societal stressor, this study provides a developmentally informed framework for understanding vulnerability to maladaptive coping in young adulthood.

## Methods

### Data

The present study was conducted as part of the IMAGEN (a European longitudinal imaging genetics study) consortium (for details, see^41^). The IMAGEN study is a multicenter project designed to investigate neurobiological, psychological, and environmental factors underlying adolescent development and risk behaviors, including substance use. Participants were assessed at multiple timepoints using a standardized protocol that includes behavioral, personality, clinical, and neuroimaging measures. The present analyses were based on a subset of participants from the IMAGEN cohort who had sufficient data to compute intra-individual personality variability and assess alcohol use outcomes. Specifically, participants were included if they provided personality data for at least two adolescent assessment waves (baseline, FU1, FU2, FU3), allowing estimation of intra-individual variability, and had available alcohol use data at one or more of the outcome timepoints (pre-pandemic FU3, COVID-T1, or COVID-T2). The IMAGEN study protocol was approved by the institutional ethics committees of King’s College London (PNM/10/11–126), the University of Nottingham (D/11/2007), Trinity College Dublin (SPREC092007-01), Technische Universität Dresden (EK 235092007), the Commissariat à l’Énergie Atomique et aux Énergies Alternatives and INSERM (2007-A00778-45), the University Medical Center at the University of Hamburg (M-191/07), and the Medical Ethics Committee of Heidelberg University (2007–024 N-MA). All procedures were conducted in accordance with the Declaration of Helsinki and relevant institutional guidelines and regulations. Written or online informed consent was obtained from all participants and, where applicable, from their parents or legal guardians before participation. Due to data protection policies, access to the IMAGEN dataset is restricted to consortium members, preventing us from sharing the dataset used in the study. The study`s analysis and design were not pre-registered.

### Participants

Participants included in the present analyses were drawn from the IMAGEN study spanning eight centers across four countries (United Kingdom, Ireland, Germany, and France^41^). Initially, 2264 healthy adolescents were enrolled in the IMAGEN study at baseline, during early adolescence (aged around 14 years), and were then followed through three subsequent waves of data collection during middle (aged around 16 years), and late adolescence (aged around 18 years), and early adulthood (aged around 22 years), respectively. Exclusion criteria for the entire IMAGEN sample included serious medical conditions, pregnancy, previous head trauma with unconsciousness, and any contraindications for functional magnetic resonance imaging (fMRI) examinations.

**Fig. 1:**
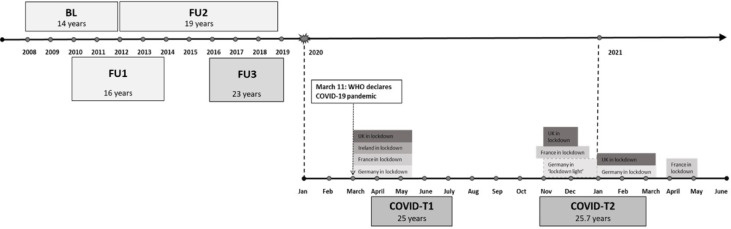
The figure depicts the data collection timeline for the present study. Data were used from six assessment time points, of which four were collected pre-COVID-19 during adolescence (BL, FU1, FU2, FU3), and two during COVID-19 (COVID-T1 and COVID-T2) at early adulthood.

Following the declaration of the coronavirus pandemic in Europe, four additional assessment waves were conducted (see Fig. 1) between April 2020 and April 2021, during young adulthood (aged around 25.5 years). The first assessment was conducted immediately after the onset of COVID-19, between April and July 2020, the second between June and July 2020, the third between November 2020 and March 2021, and the fourth between March and June 2021. This resulted in a total of eight assessment time points, with four measurements before the COVID-19 pandemic (here referred to as pre-COVID-19 timepoints) and four measurements during the pandemic (here referred to as the COVID-19 timepoints). For the pre-COVID-19 timepoints, *N* = 2264 were included at early adolescence (BL, see Fig. 1), *N* = 1768 at middle adolescence (FU1), *N* = 1585 at late adolescence (FU2), and *N* = 1377 at early adulthood, (FU3). During COVID-19, the sample sizes were *N* = 563 at the first, *N* = 342 at the second, *N* = 324 at the third, and *N* = 105 at the fourth timepoint. Because not all centers and participants from the BL to FU3 assessments took part in the assessments during COVID-19, the sample size is much lower compared to periods before COVID-19.

For the current study, we selected a subset of assessment samples from the IMAGEN cohort, including only participants who had complete data on personality factors and alcohol use measures across all pre-COVID-19, as well as alcohol use data for any of the COVID-19 timepoints (i.e., complete-case analysis). This resulted in a sample size of *N* = 968 (*n* = 527 females and *n* = 441 males) for all pre-COVID-19 timepoints, and *N* = 396 (*n* = 239 females and *n* = 157 males) for the first and *N* = 244 (*n* = 155 females, *n* = 89 males) for the third COVID-19 timepoints. We did not include data from the second COVID-19 timepoint due to the absence of relevant alcohol use measures. Additionally, we excluded data from the fourth COVID-19 timepoint because the participant numbers were insufficient to achieve adequate statistical power for our analyses. Therefore, for the COVID-19 analyses, we focused only on data from the first (collected immediately after the onset of the pandemic) and the third (collected a few months after, during the pandemic) COVID-19 timepoints, which are referred to as COVID-T1 and COVID-T2, respectively (for an overview, see Fig. 1).

Additionally, we conducted a post-hoc statistical power analysis using G*Power 3.1^44^ to confirm that our sample size was sufficient for the analyses conducted. Effect sizes of non-linear Generalized Additive Mixed Models (GAMMs, in the present study) can vary across predictor variable ranges, making formal effect size estimation challenging. Additionally, we conducted a post-hoc statistical power analysis using G*Power 3.1^44^ to confirm that the available sample size was sufficient for the planned analyses. Because effect sizes in non-linear Generalized Additive Mixed Models (GAMMs) can vary across the range of predictor values and are not directly summarized by a single standardized coefficient, formal effect-size estimation is challenging. We therefore used a medium effect size of f² = 0.15 as a pragmatic benchmark for the power analysis. This choice was based on Cohen’s conventional effect-size classification and was intended to provide a conservative reference point for detecting effects of practical relevance in the context of multiple predictors and longitudinal data. Using α = .05 and f² = 0.15, the analyses indicated adequate statistical power for both the pre-COVID-19 sample (N = 968) and the COVID-19 sample used in the main analyses (N = 244), supporting the adequacy of the sample size for the models performed.

### Design

To analyze our hypotheses, we used Generalized Additive Mixed Models (GAMMs). GAMMs extend linear mixed-effects models (LMEs) and are particularly useful for modeling longitudinal data without assuming linearity. Unlike traditional growth curve models, GAMMs do not impose rigid constraints on the data, instead fit smooth trajectories based on input data. GAMMs are well-suited for analyzing longitudinal repeated measures data because they account for within-subject correlations. Additionally, GAMMs can accommodate random effects and interactions between variables. Smooth terms in the GAMMs are characterized by their estimated degrees of freedom (EDF), which indicate the complexity of the fitted function. An EDF value of approximately 1 suggests a linear relationship between the predictor and outcome, whereas higher EDF values indicate increasing non-linearity. Thus, EDF provides an estimate of the flexibility of the smooth term, with larger values reflecting more complex, non-linear associations. Model fit for the GAMMs was evaluated using standard fit indices implemented in the mgcv package. Specifically, model fit was assessed using the generalized cross-validation (GCV) score and the Akaike Information Criterion (AIC), which were used to evaluate and compare alternative model specifications. In addition, deviance explained was examined as an indicator of the proportion of outcome variability accounted for by the model. Model diagnostics further included visual inspection of residuals to assess distributional fit, potential deviations from homoscedasticity, and other violations of model assumptions. For detailed information on GAMMs, refer to Wood^45^. We used the R package *mgcv*^46^ to implement our GAMMs.

To test Hypothesis 1, which predicted that variability in anxiety sensitivity and impulsivity would be associated with alcohol use during the pandemic and more strongly in females, we fitted GAMMs with personality variability as predictors of alcohol use at each timepoint (FU3, COVID-T1, COVID-T2), including sex as a covariate and testing interactions between sex and the respective predictors.

To test Hypothesis 2, which predicted that variability in openness would be more strongly associated with pre-pandemic alcohol use, we fitted GAMMs with openness variability as a predictor of alcohol use at FU3 and compared patterns across timepoints.

To test Hypothesis 3, which predicted that country-level lockdown stringency would moderate associations between personality variability and alcohol use, we included interaction terms between personality variability and lockdown stringency in the GAMMs to examine whether associations differed as a function of restriction severity.

The predictor variables were intra-individual variability scores of personality traits (NEO-FFI and SURPS). Interactions were tested between these predictors and sex to examine whether associations with alcohol-use outcomes differed between males and females. In GAMMs, this was implemented by estimating separate smooth functions for each level of the categorical variable. We also employed a priori treatment contrasts to examine interaction effects between sex (added as a covariate in our models) and our predictor variables. It is important to clarify that when modeling the interactions between the covariates (coded as factors) and predictors using GAMMs, the output does not directly summarize interaction effects. Instead, GAMMs produce separate smooths for each level of the covariate factor. To assess whether there was an interaction between sex (assessed via two labels, male and female) and our predictor variables, we followed the approach outlined by Jones et al.^9^,. We refitted our models with sex coded as an “ordered factor” variable and included it as an interaction term in these refitted models to generate a *difference smooth*^9^. A significant *difference smooth* indicates that the two sexes follow distinct trends. This approach is analogous to including an interaction term in traditional LME models^9^. We present interaction effects of sex only for those personality factors that demonstrated significance in our models. Personality variability was operationalized as the intra-individual variance of repeated personality trait scores across the four pre-COVID-19 assessment waves. This approach captures the extent to which individuals fluctuate around their typical trait levels over time. This operationalization differs from modeling approaches such as latent growth curve models or latent change score models, which are designed to estimate systematic directional change across time. In contrast, the present approach focuses on variability as an indicator of the stability versus fluctuation of personality traits across adolescence. Accordingly, the present study conceptualizes personality development in terms of intra-individual variability, capturing differences in the consistency of trait expression over time. This measure does not distinguish between systematic trends and short-term fluctuations, and therefore reflects one aspect of personality development rather than directional change.

To examine potential selective attrition, we compared participants retained in the COVID-19 analyses with those lost to follow-up on key baseline variables assessed at pre-pandemic early adulthood (FU3). Specifically, we compared groups on sex and country using chi-square tests, and on continuous variables including alcohol-use frequency and personality traits (NEO-FFI and SURPS subscales) using independent-samples t-tests. All tests were two-tailed with a significance threshold of α = 0.05.

### Data acquisition and materials

#### Personality

Personality was measured at all pre-COVID-19 assessment timepoints, using two standardized personality questionnaires: the Neuroticism-Extraversion-Openness five-factor inventory (NEO-FFI) and the substance use risk profile scale (SURPS).

The NEO-FFI^47^ is a widely used and well-validated personality measure with high test-retest reliabilities, ranging from 0.86 to 0.90^47,48^. The NEO-FFI comprises a total of 60 items, divided into five subscales: openness, extraversion, neuroticism, conscientiousness, and agreeableness, each containing twelve items. Each item is rated on a 5-point Likert scale, with responses ranging from 0 (strongly disagree) to 4 (strongly agree). The scores on all subscale items are averaged to yield a mean subscale score. Each participant provided information on five subscale scores for the NEO-FFI, where a higher mean score corresponds to a higher level of the factor, while a lower score corresponds to a lower level of the factor.

The SURPS is a 23-item screening instrument that examines four distinct dimensions of personality linked to the risk of developing addictive disorders^49^. The 23 items are divided into a total of four subscales, namely impulsivity, sensation seeking, anxiety sensitivity, and hopelessness, each comprising 5–7 items respectively. Each item is rated on a 4-point Likert scale, with responses ranging from “strongly disagree” (1) to “strongly agree” (4). Subscale scores were calculated by averaging scores on items belonging to each subscale. In total, each participant provided information on four subscale scores for the SURPS, where a higher mean score on a subscale corresponds to a higher level of the factor while a lower score corresponds to a lower level of the factor. The SURPS has demonstrated acceptable to good psychometric properties in adolescent and adult samples, including evidence for internal consistency, test-retest reliability, and concurrent and predictive validity for substance-use-related outcomes^50–52^.

#### Alcohol use

Alcohol use was assessed at all pre-COVID-19 timepoints, and immediately after the onset of (COVID-T1) and during COVID-19 (COVID-T2), using the Alcohol Use Disorders Identification Test (AUDIT). The AUDIT is a 10-item screening instrument for hazardous and harmful alcohol consumption^53^, which has shown good reliability and validity across multiple populations. Previous studies have reported good internal consistency for the AUDIT, with Cronbach’s α values around 0.80, as well as high test-retest reliability for the total score, with intraclass correlation coefficients reported up to 0.95^54–56^. It comprises three subscales: alcohol-use frequency, dependence symptoms and harmful alcohol use, and an audit total score. For the purpose of our study, we used the first subscale, i.e., alcohol-use frequency. This subscale comprises three items, asking respondents about their quantity and frequency of drinking, including an item about binge drinking. For all three items, the responses range from 0 to 4. The subscale score was calculated by taking a sum of the three item scores, with higher values representing a higher quantity and frequency of alcohol consumption and vice versa. This AUDIT subscale score of alcohol-use frequency was our main outcome variable.

### Statistical analysis

#### Data pre-processing and quality control

All statistical analyses were conducted using the statistical software R^57^. Data were initially inspected for outliers and assessed for normal distribution though visual inspection of box plots and histograms (see Supplement). Given the unequal distribution of participants across centers, we adjusted for country and regional effects by grouping centers in three levels for the variable: Germany, France, and the UK + Ireland (for a detailed overview, see Suppl. Materials, Table 1). We applied a treatment contrast to this variable, using Germany as the reference level of comparisons. We also applied a treatment contrast to the variable sex in order to compare differences between females and males.

#### Changes in personality

**Fig. 2:**
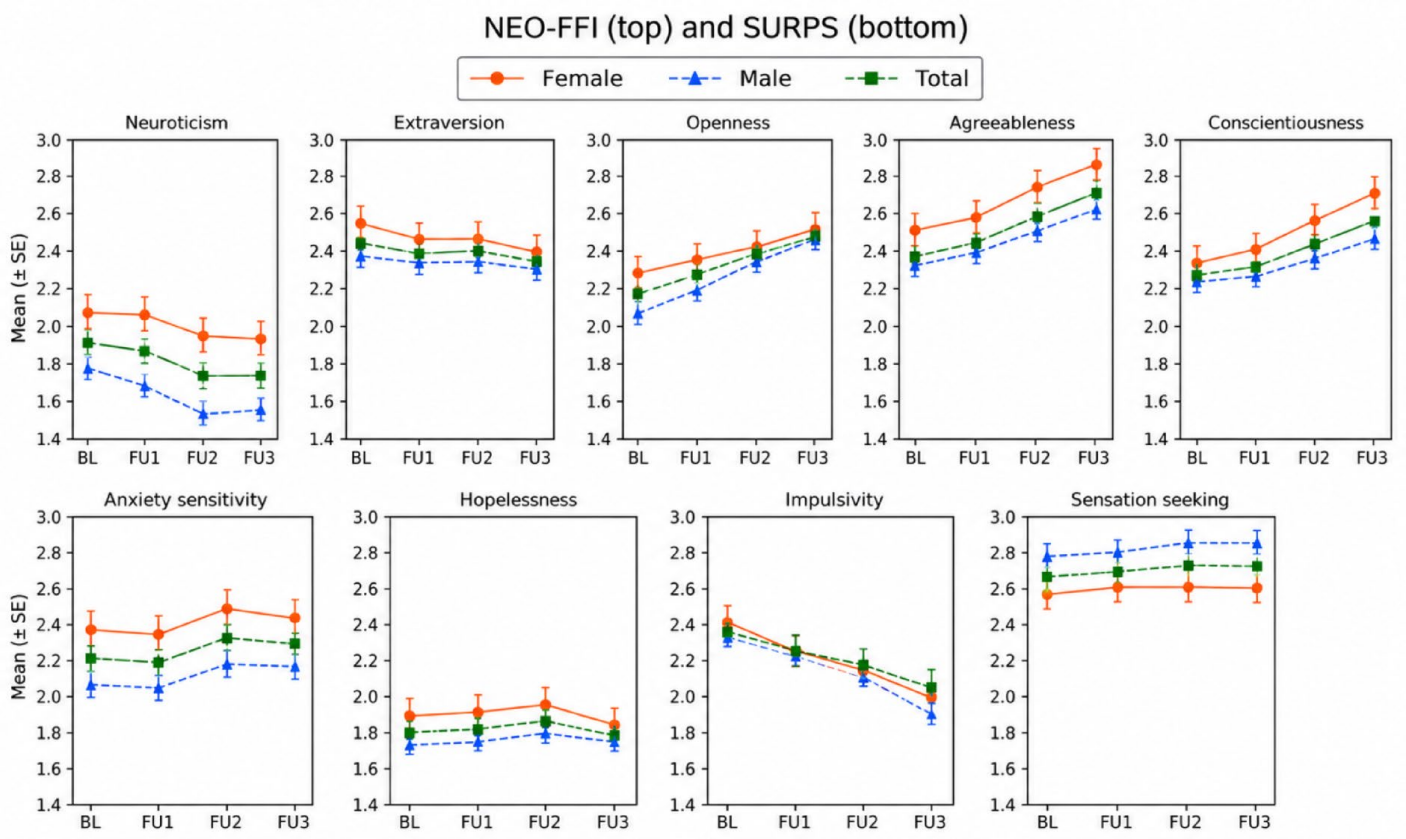
Descriptive statistics of personality factors assessed with NEO-FFI and SURPS across pre-COVID and COVID timepoints.

**Fig. 3:**
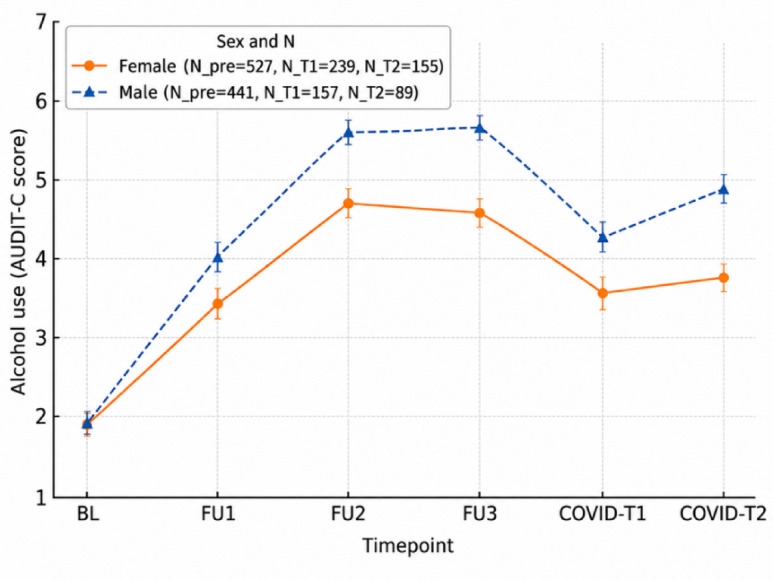
Descriptive statistics of alcohol use across pre-COVID and COVID timepoints.

As our study employs a longitudinal design with personality assessed at all four pre-COVID-19 timepoints, each participant in our dataset provided subscale scores for both personality questionnaires four times, resulting in four scores for each subscale (5 subscales scores x 4 timepoints for NEO-FFI and 4 subscales scores x 4 timepoints for SURPS). We operationally defined changes in personality factors during adolescence as variance across these four personality scores, where a higher variance indicated greater changes. This resulted in a total of nine variance scores for each participant: five for the NEO-FFI subscales and four for the SURPS subscales (see Figs. 2 and 3). These variance scores served as the primary predictor variables in all statistical models. Variance calculations were performed using base-R’s var() function.

#### Changes and relative changes in alcohol-use frequency scores

To first examine alcohol use and the changes throughout the pre- and COVID-19 timepoints in our sample (see Fig. 3), we conducted two one-way repeated measures analyses of variance (ANOVAs). In the first ANOVA, we compared alcohol use frequency scores across pre-COVID-19 timepoints (*N* = 968), with timepoint as the within-subjects factor with four levels (BL, FU1, FU2 and FU3). The second ANOVA was conducted to assess alcohol use frequency scores across both pre- and COVID-19 timepoints, resulting in a reduced sample size (*N* = 244) comprising participants with complete data across all timepoints (i.e., complete-case analysis). Timepoint was coded as the within-subjects factor. Due to violations of the homogeneity of variance assumption, Greenhouse Geiser adjustments were applied to both ANOVAs.

Additionally, we calculated relative changes in alcohol-use frequency scores to analyze whether changes in personality factors predicts changes in alcohol-use frequency scores from pre-COVID-19 to COVID-19. We calculated two relative change scores using the formula $\:\frac{TP2-TP1}{TP1}\:$; to examine changes in alcohol use from pre-COVID-19 (FU3) to immediately after the onset of COVID-19 (COVID-T1), and from pre-COVID-19 (FU3) to during COIVD-19 (COVID-T2).

To analyze whether changes in personality factors during adolescence (pre-COVID), modelled as variation across corresponding timepoints, would predict alcohol use in young adults immediately after the onset of (COVID-T1) and during (COVID-T2) COVID-19, we built two GAMM models. Changes in the personality factors of the NEO-FFI and SURPS were coded as smooth predictors in both models. Alcohol-use frequency scores at COVID-T1 was the outcome variable for Model 1 and alcohol-use frequency scores at COVID-T2 was the outcome variable for Model 2.

To analyze whether changes in personality factors during adolescence would predict change in alcohol use from pre-COVID-19 to COVID-T1, and pre-COVID-19 to COVID-T2, we built two additional GAMMs. Changes in personality traits factors of the NEO-FFI and SURPS were coded as smooth predictors in both models. Relative changes in alcohol-use frequency scores from pre-COVID-19 to COVID-T1 was the outcome variable for Model 3 and relative changes in alcohol-use frequency scores from pre-COVID-19 to COVID-T2 was the outcome variable for Model 4.

To analyze whether the significant associations between changes of personality factors and alcohol use observed pre-COVID-19 are similar to those observed immediately after the onset of (COVID-T1) and during COVID-19 (COVID-T2), we ran a fifth model. This Model 5 was used to check the pre-COVID-19 associations between changes in personality factors from BL to FU2 and alcohol use during early adulthood (FU3). For this, changes in the personality factors of the NEO-FFI and SURPS were used as smooth predictors and alcohol-use frequency scores assessed pre-COVID-19 FU3 was the outcome variable.

In all our models, sex and country were added as covariates. A random effect of participants (i.e., patients’ ID) was also added to account for repeated measures.

### Attrition analysis

The analytic sample included *N* = 968 participants with complete pre-COVID-19 data, of whom *N* = 396 were retained at COVID-T1 and *N* = 244 at COVID-T2. To examine potential selective attrition, we compared participants retained at each COVID-19 timepoint with those lost to follow-up on key baseline variables assessed at pre-pandemic early adulthood (FU3). Independent-samples t-tests were conducted for continuous variables (alcohol-use frequency and personality traits), and chi-square (χ²) tests were used for categorical variables (sex and country). Effect sizes are reported as Cohen’s d for t-tests and Cramér’s V for chi-square tests. At COVID-T1, there were no significant differences between retained and non-retained participants in baseline alcohol-use frequency or personality traits (all |t| < 1.65, all *p* > 0.10, Cohen’s d < 0.15). Chi-square tests indicated no significant differences in the distribution of sex or country (all χ² < 3.00, all *p* > 0.05, Cramér’s V < 0.08). At COVID-T2, results were comparable. No significant differences were observed between retained and non-retained participants in baseline alcohol-use frequency or personality measures (all |t| < 1.80, all *p* > 0.05, Cohen’s d < 0.20). The proportion of female participants was slightly higher among those retained; however, this difference was not statistically significant (χ² < 3.50, *p* > 0.05, Cramér’s V < 0.10). No significant differences were observed for country distribution (χ² < 4.00, *p* > 0.05, Cramér’s V < 0.10). Results of attrition analyses show that attrition across COVID-19 timepoints was not systematically associated with key baseline variables, providing no evidence for substantial selective attrition bias.

## Results

### Sample description

Data from a total of 968 participants were analyzed (*n* = 968, F = 54.4%), 49.8% were from Germany (F = 54.2%), 37.7% from UK and Ireland (F = 53.7%) and 12.5% were from France (F = 57.9%).

Alcohol-use frequency scores significantly increased steadily from early adolescence (BL; *M* = 0.93, *SD* = 1.35) to late adolescence (FU2; 3.74, *SD* = 2.37) and remained stable from late adolescence (FU2) until early adulthood (FU3; *M* = 3.67, *SD* = 2.08). There was a notable decrease at the onset of COVID-19, i.e., COVID-T1 (*M* = 2.65, *SD* = 2.23), followed by a subsequent increase up to COVID-T2 (*M* = 2.83, *SD* = 1.98) (see Fig. 2). The mean relative changes in alcohol-use frequency scores were not significant (from FU3 to COVID-T1: *M* = −0.19, *SD* = 0.484, *p* > 0.05; from FU3 to COVID-T2: *M* = −0.12, *SD* = 0.466, *p* > 0.05).

**Table 1:** Results of repeated measures ANOVA, comparing alcohol use across pre-COVID-19 timepoints (*n* = 968) and pre and COVID-19 timepoints (*n* = 244).

Within Subjects effect	*n*	df	F	*P*	η^2^_G_
pre-COVID-19 timepoints	*968*	2.78	845.53	< 0.05	0.279
		2683.96			
pre- and COVID-19 timepoints	*244*	4.05	143.71	< 0.05	0.217
		983.32			

###  Changes in alcohol-use frequency scores

The pre-COVID-19 one-way repeated measures ANOVA revealed a significant main effect of time on alcohol-use frequency scores [*F* (2.78, 2683.96) = 845.53, *p* < 0.05]. Bonferroni corrected post-hoc comparisons showed significant differences between all pairwise comparisons except between FU2 and FU3, suggesting an increase in alcohol-use score at each subsequent timepoint (see Table 1). The second ANOVA, conducted to compare alcohol-use frequency scores across the pre- and post-COVID-19 timepoints, also yielded a significant main effect of time [*F* (4.05, 983.32) = 143.71, *p* < 0.05]. Post-hoc pairwise comparisons using Bonferroni corrections indicated significant differences between all pairs of timepoints, except between FU2 and FU3, meaning an increase in alcohol-use frequency score at each subsequent pre-COVID-19 timepoint, with a decrease observed from pre-COVID-19 to COVID-T1 and a subsequent increase observed from COVID-T1 to COVID-T2.

### Model 1 & 2. changes of personality factors during adolescence, pre-COVID-19, as predictor of alcohol use in young adulthood at COVID-T1 and COVID-T2

In Model 1, changes in personality factors measured with the NEO-FFI was not a significant predictor of alcohol-use frequency scores at COVID-T1. However, changes in anxiety sensitivity were not significant (EDF = 1.300, *p* = 0.08).

In Model 2, changes in the personality factor of anxiety sensitivity was associated with higher alcohol-use frequency at COVID- T2 (EDF = 1.862, *p* < 0.05). There was no significant interaction between gender and anxiety sensitivity (*p* > 0.05). However, the smooth was significant for females (EDF = 1.000, *p* < 0.05) as compared to males (EDF = 1.000, *p* > 0.05), i.e., both male and female participants followed a similar trajectory but anxiety sensitivity was a significant predictor of alcohol use only in females at this timepoint.

### Model 3 & 4. changes of personality factors during adolescence, pre-COVID-19, as a predictor of change in alcohol use from pre-COVID-19 to COVID-T1, and pre-COVID-19 to COVID-T2

In both Model 3 and Model 4, variability in personality traits was not significantly associated with changes in alcohol-use frequency (all p-values > 0.05), indicating that personality variability did not predict short-term or longer-term changes in alcohol use during the pandemic. These findings suggest that adolescent personality variability did not predict short-term or longer-term changes in alcohol use during the pandemic.

### Model 5. changes of personality factors during adolescence, pre-COVID-19, as predictor of alcohol use pre-COVID-19, in early adulthood

Model 5 revealed that changes in the personality factor of openness was a significant predictor of higher alcohol-use frequencyat early adulthood (FU3; EDF = 1.000, *p* < 0.05). This was significant for both males (EDF = 1.000, *p* < 0.05) and females (EDF = 1.000, *p* < 0.05). However, the interaction between openness and gender was not significant (*p* > 0.05), meaning that no gender differences could be detected.

**Fig. 4:**
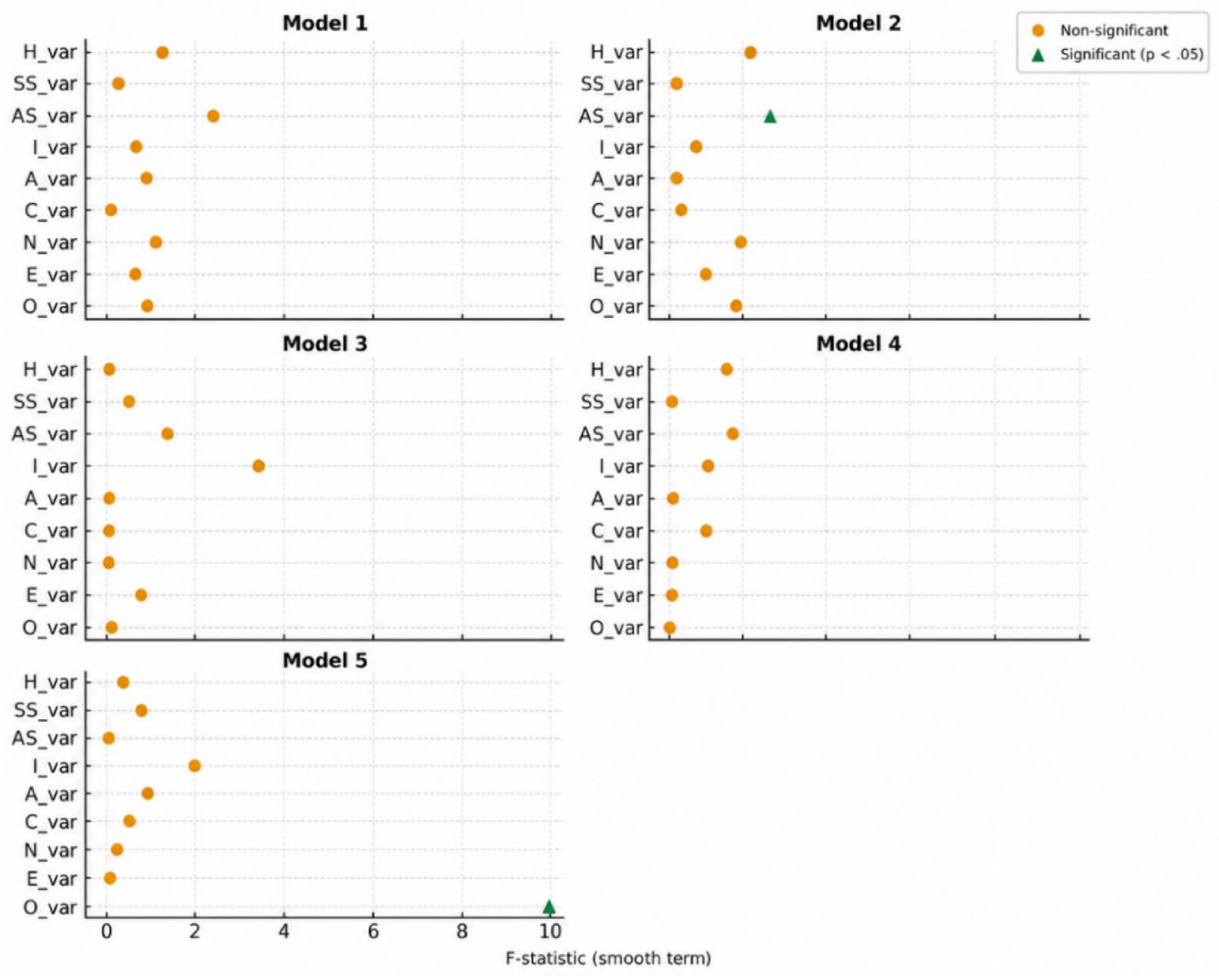
Smooth-term effects for personality variability predictors across generalized additive mixed models (GAMMs). The figure displays F-statistics for smooth terms representing intra-individual variability in personality traits (O_var = Openness, E_var = Extraversion, N_var = Neuroticism, C_var = Conscientiousness, A_var = Agreeableness, I_var = Impulsivity, AS_var = Anxiety Sensitivity, SS_var = Sensation Seeking, H_var = Hopelessness) across five GAMMs predicting alcohol-use frequency at different time points (Models 1–5). Each row corresponds to one model, and each dot represents the smooth-term estimate for one predictor. Orange circles indicate non-significant effects (*p* > 0.05), while green triangles indicate significant smooth terms (*p* < 0.05). Predictor order is held constant across panels to allow for direct visual comparison. Significant predictors include anxiety sensitivity variability at COVID-T2 (Model 2) and openness variability at pre-pandemic FU3 (Model 5). Full numerical model summaries, including parametric coefficients and smooth-term statistics, are provided in Supplementary Table S1.

**Fig. 5:**
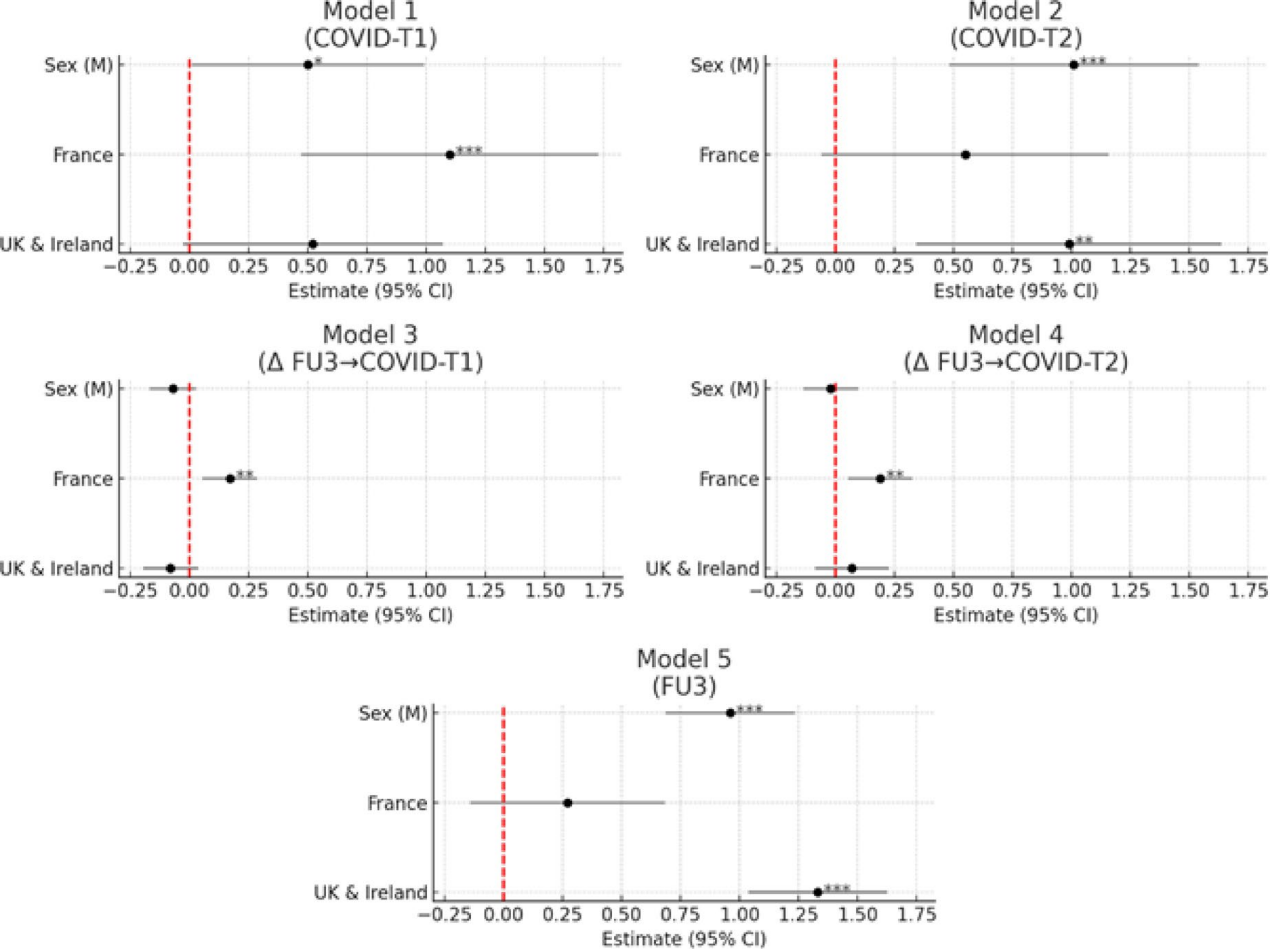
Forest plots of parametric (linear) coefficients for sex and country across five generalized additive mixed models (GAMMs) predicting alcohol-use outcomes. Each panel corresponds to one model: COVID-T1 (Model 1), COVID-T2 (Model 2), change from FU3→COVID-T1 (Model 3), change from FU3→COVID-T2 (Model 4), and pre-pandemic FU3 alcohol use (Model 5). Points represent estimated regression coefficients with 95% confidence intervals for sex (male vs female) and country (France; UK & Ireland, reference = Germany). Statistical significance is indicated by asterisks: *p < .05, **p < .01, ***p< .001; coefficients without an asteriks were not statistically significant (*p* ≥ .05).

A summary of the generalized additive mixed models can be found in Figs. 4 and 5.

The results of additional pre-COVID-19 models are reported in the supplementary materials (see Suppl. Materials).

## Discussion

The present study aimed to examine whether intra-individual variability in personality traits during adolescence predicts alcohol use in young adulthood, both prior to and during the COVID-19 pandemic, and whether these associations are moderated by sex and contextual factors. Based on theoretical perspectives such as the maturity principle and the social investment principle, as well as frameworks linking personality instability to maladaptive coping, we hypothesized that greater variability in traits related to emotional reactivity and impulsivity would be associated with higher alcohol use, particularly under conditions of stress. Importantly, variability in certain personality traits may reflect affective dysregulation, defined as difficulties in maintaining stable and adaptive emotional responses over time. Individuals with affective dysregulation often experience heightened emotional reactivity and reduced capacity to regulate negative affect, which has been linked to maladaptive coping strategies such as substance use^23,24^. Within this framework, intra-individual variability in traits such as anxiety sensitivity may serve as an indicator of unstable emotional processing and increased vulnerability to stress-related drinking.

Consistent with our first hypothesis, greater variability in anxiety sensitivity was associated with higher alcohol use during the later phase of the pandemic (COVID-T2). This effect was primarily observed in females and followed a non-linear pattern, suggesting that higher levels of variability were linked to increased alcohol consumption. In contrast, no significant associations were observed at COVID-T1, indicating that personality-related risk may not manifest immediately following the onset of a stressor. Anxiety sensitivity is a known transdiagnostic risk factor for internalising symptoms and maladaptive coping^58^. Variability in this trait may reflect instability in emotional regulation capacities, which are still consolidating during adolescence^59,60^. Adolescents who fluctuate strongly in anxiety sensitivity may experience more unpredictable emotional responses, increasing the likelihood of using alcohol as a coping strategy when facing sustained stressors. Importantly, this effect was not present at COVID-T1, suggesting a delayed vulnerability. The early months of the pandemic were characterized by reduced alcohol access and strict mobility restrictions, which may have attenuated drinking behaviour regardless of underlying risk processes.

The current findings align with prior evidence linking trait instability to affective dysregulation and avoidance coping strategies^23,24,61–64^. In the context of prolonged stressors like the pandemic, adolescents with unstable anxiety sensitivity may lack consistent adaptive coping strategies, increasing the likelihood of using alcohol to manage distress. Importantly, this effect emerged despite controlling for pre-pandemic drinking, underscoring its unique contribution during crisis contexts.

Partially supporting our second hypothesis, greater variability in openness was associated with higher alcohol use prior to the pandemic (FU3). This finding is consistent with previous research linking openness to sensation seeking and socially motivated drinking. In contrast, variability in other personality traits did not significantly predict pre-pandemic alcohol use. This pattern suggests that personality variability may relate to different drinking motives depending on the trait domain. While openness may be linked to exploratory and socially driven alcohol use, variability in anxiety sensitivity appears more relevant for stress-related or coping-motivated drinking, highlighting the domain-specific nature of these effects. Pre-pandemic, intra-individual changes in openness were significantly linked to alcohol use, consistent with prior work showing that openness is associated with sensation seeking and socially motivated drinking^13,65,66^. However, during the pandemic, variability in anxiety sensitivity - a trait more strongly linked to internalising distress - was the dominant predictor, especially for females. This shift in predictive patterns reflects broader changes in drinking motives during the pandemic^67^: alcohol was less likely consumed for enhancement or social reasons, and more for coping with negative affect. Openness is often associated with externalising tendencies and positive reinforcement motives (e.g., novelty seeking, peer engagement)^68^, while anxiety sensitivity is associated with negative reinforcement (e.g., drinking to alleviate distress)^69–71^. These findings support the view that stress-related drinking is governed by distinct personality mechanisms and highlight the importance of context in understanding substance use pathways.

The findings provide partial support for the hypothesis that contextual factors moderate personality–alcohol associations. Specifically, the predictive role of personality variability differed between pre-pandemic and pandemic periods. During the pandemic, variability in anxiety sensitivity emerged as the key predictor, whereas openness was more relevant prior to the pandemic. This shift is consistent with research showing that drinking motives changed during COVID-19, with reduced emphasis on social/enhancement motives and increased reliance on coping-related drinking. Thus, the results highlight that personality-related risk is context-dependent, with different traits becoming relevant depending on environmental demands and stress exposure.

The observed association between anxiety sensitivity and alcohol use emerged only at COVID-T2, not immediately after the initial lockdown (COVID-T1). This temporal lag suggests a delayed vulnerability effect. Such latency is consistent with research on “sleeper effects” following large-scale stressors, where mental health or behavioural consequences unfold over time rather than immediately^39,72^. Early in the pandemic, environmental constraints (e.g., limited alcohol access, mobility restrictions)^40,73^ may have suppressed drinking behaviours even among at-risk individuals. As restrictions eased and the psychological toll accumulated, individuals with unstable anxiety sensitivity may have turned to alcohol as a maladaptive coping strategy^35,37^. Future studies should model dynamic interactions between personality factors, policy environments, and coping trajectories to better understand these time-sensitive effects.

The gender-specific effects further suggest that females with fluctuating anxiety sensitivity may be particularly vulnerable. Prior studies have shown steeper increases in alcohol consumption and related harms among females during the pandemic, potentially due to greater exposure to pandemic-related stressors, higher internalising symptoms, and reduced access to social support^74–76^. Given that reassurance and social connection buffer anxiety sensitivity, the social isolation during COVID-19 may have disproportionately affected females, contributing to maladaptive coping via alcohol use.

### Limitations

While this study provides important insights, several limitations should be noted as well.

First, personality variability was operationalized as the intra-individual variance across repeated assessments, which captures fluctuations in trait expression but does not distinguish between systematic developmental change (e.g., increases or decreases over time) and short-term variability. Future studies could complement this approach by using latent growth curve models or latent change score models to differentiate between directional change and instability in personality development.

Second, alcohol use was assessed using self-report measures, which may be subject to recall bias or social desirability effects. Future research could incorporate multi-method assessments, such as ecological momentary assessment or biological indicators, to obtain more fine-grained and objective measures of substance use. Third, although we examined the moderating role of sex and country-level context, other potentially relevant factors were not included. For example, individual differences in coping motives, stress exposure, or mental health symptoms during the COVID-19 pandemic may further explain variability in alcohol-use outcomes. Future research should investigate these mechanisms to better understand how personality variability translates into behavioral risk.

### Conclusion and future outlook

This study demonstrates that intra-individual variability in personality traits during adolescence, particularly anxiety sensitivity, significantly predicts alcohol use during, but not immediately after, the onset of a large-scale societal stressor. Moreover, different personality factors were relevant for predicting alcohol use before versus during the pandemic, suggesting that the contextual salience of certain traits shifts under stress. Our results underscore the importance of considering developmental trajectories and personality instability - not only trait levels - when identifying individuals at risk for maladaptive substance use.

These findings have direct implications for post-pandemic prevention: screening tools that identify individuals with fluctuating anxiety sensitivity during adolescence may help target youth at elevated risk for coping-related drinking. Gender-sensitive approaches are also warranted, given the stronger effects observed in females.

Future research should explore whether these effects persist beyond the pandemic and whether personality stability can be enhanced through intervention. The COVID-19 crisis serves as a natural experiment illustrating how internal developmental factors interact with external disruptions to shape health-risk behaviours. Understanding these interactions is crucial for building more resilient developmental pathways - and for preparing for future crises.

## Supplementary Information

Below is the link to the electronic supplementary material.

Supplementary Material 1

## Data Availability

Analyzed and reported data of this study is available upon request to the corresponding author.

## Declarations

### Transparency declaration

We, the authors and co-authors, declare that this work is an honest, accurate, and transparent account of the study being reported. No important aspects of the study have been omitted and any discrepancies from the study as planned have been explained.

### Analytic code availability

Analytic code of this study is available upon request to the corresponding author.

### Research material availability

Research Material of this study is available upon request to the corresponding author.
