# Supplementary material for: Personality variability during development predicts alcohol use before and during the COVID-19 pandemic
Source: Sci Rep. 2026 Aug 14;16:25517. doi: 10.1038/s41598-026-63441-y (PMC13476260; doi:10.1038/s41598-026-63441-y)
Supplement: Supplementary file 1 [file 41598_2026_63441_MOESM1_ESM.docx]

**Supplemental Information**

**1 Supplemental Methods**

**1.1 Pre-COVID-19 analysis**

Additional analyses were performed to check whether changes in personality traits during adolescence a) cross-sectionally predict alcohol use at late adolescence, and b) longitudinally predict an increase in alcohol use from middle adolescence to early adulthood and from late adolescence to early adulthood.

*1.1.1 Changes in personality:*

For the cross-sectional pre-COVID-19 analysis, we operationally defined changes in personality traits as variance in personality trait scores across the four pre-COVID-19 timepoints. For this, we only calculated variance across the first three timepoints, from early to late adolescence (See suppl. table 4). These variance scores were the predictor variables for the supplementary analyses.

*1.1.2 Relative Change in* *alcohol-use frequency scores:*

For the longitudinal pre-COVID-19 analysis we calculated two relative change scores, from middle adolescence to early adulthood, and late adolescence to early adulthood (See suppl. table 2). These relative change scores were the outcome variables for the respective longitudinal analyses.

*1.1.3 Modelling and hypothesis testing:*

Generalized Additive Mixture Models (GAMMs) were used to carry out the supplementary analyses. We built three models, model 6,7, and 8. Model 6 was used to conduct additional pre-COVID-19 cross-sectional analysis, with changes in personality traits as predictor of alcohol-use frequency during late adolescence. Model 7 and 8 were used to carry out the additional pre-COVID-19 longitudinal analysis, with changes in personality traits as predictor variable and *Relative change in alcohol-use frequency scores,* from middle adolescence to early adulthood, and from late adolescence to early adulthood, as outcome variables for model 7 and 8 respectively. We also used a priori contrasts to check for interaction effects between our covariates and predictor variables (For a summary of these models, see suppl. Table. 3).

**2 Supplemental results**

**2.1 Pre-COVID-19 analysis**

*Model 6. Changes of personality traits* *during adolescence as predictor of alcohol-use frequency scores during late-adolescence.* No significant effect of changes in personality traits was found on alcohol-use frequency scores during late-adolescence (see suppl. Table 3).

Model 7. *Changes of personality traits during adolescence as predictor of relative change in alcohol-use frequency* *scores from middle-adolescence to early-adulthood.* Variance in personality did not predict change alcohol-use frequency scores from middle-adolescence to early-adulthood. However, variance in the trait of impulsivity (EDF = 1.000, p = 0.05) was approaching significance (see suppl. Table 3).

Model 8*. Changes of personality traits during adolescence as predictor of relative change in alcohol-use frequency scores from late-adolescence to early-adulthood.* Variance in the personality trait of conscientiousness significantly predicted relative changes in alcohol-use frequency scores from late-adolescence to early-adulthood, following a nonlinear trend (EDF = 6.766, p < 0.05). There was a significant interaction between conscientiousness and gender (p< 0.05) where the effect of conscientiousness on the outcome was significant and non-linear for male participants (EDF =7.919, p < 0.05) as compared to female participants (EDF = 1.001, p > 0.05) who followed linear trend that was not significant (see suppl. Table 3).

**Diagnostic Plots**

# 1 Alcohol-use frequency

BL

FU1

FU2

FU3

# 2 Neuroticism

BL

FU1

FU2

FU3

# 3 Extraversion

BL

FU1

FU2

FU3

# 4 Openness

BL

FU1

FU2

FU3

# 5 Agreeableness

BL

FU1

FU2

FU3

# 6 Conscientiousness

BL

FU1

FU2

FU3

# 7 Hopelessness

BL

FU1

FU2

FU3

# 8 Anxiety sensitivity

BL

FU1

FU2

FU3

# 9 Impulsivity

BL

FU1

FU2

FU3

# 10 Sensation seeking

BL

FU1

FU2

FU3

**Supplementary Tables:**

| **Country** | **gender** | **Early-**  **Adolescence** | **Middle-**  **Adolescence** | **Late- Adolescence** | **Early-Adulthood** | **COVID-T1** | **COVID- T2** |
| --- | --- | --- | --- | --- | --- | --- | --- |
|  |  | *n* | *n* | *n* | *n* | *n* | *n* |
| Germany | F | 261 | 261 | 261 | 261 | 84 | 54 |
|  | M | 221 | 221 | 221 | 221 | 52 | 27 |
| United Kingdom + Ireland | F | 196 | 196 | 196 | 196 | 102 | 52 |
|  | M | 169 | 169 | 169 | 169 | 63 | 25 |
| France | F | 70 | 70 | 70 | 70 | 53 | 49 |
|  | M | 51 | 51 | 51 | 51 | 42 | 37 |

Table 1. Participant distribution across countries. F: Female, M: Male

|  | **Gender** | *n* | **Early-adulthood – Middle-adolescence/ Middle-adolescence** | |  | **Early-adulthood – Late-adolescence/ Late-adolescence** | |
| --- | --- | --- | --- | --- | --- | --- | --- |
|  |  |  | *M* | *SD* |  | *M* | *SD* |
| Alcohol use frequency |  |  | 0.95 | 1.54 |  | 0.18 | 0.76 |
|  | *F* | *527* | 0.83 | 1.36 | *527* | 0.17 | 0.74 |
|  | *M* | *441* | 1.09 | 1.73 | *441* | 0.19 | 0.79 |

Table 2. Relative change in alcohol-use frequency across pre-COVID timepoints

|  | **Parametric coefficients** |  |  |  |  | **Smooth terms** | |  |
| --- | --- | --- | --- | --- | --- | --- | --- | --- |
|  | **Estimate** | **Std. Error** | **t-value** | **p-value** |  | **EDF** | **F-stat** | **p-value** |
| Model 6 | Alcohol use frequency (Late-adolescence) ~ s(O_var) + s(E_var) + s(N_var) + s(C_var) + s(A_var) + s(I_var) + s(AS_var) + s(SS_var) + s(H_var) + gender + country | | | | | | |  |
|  |  |  |  |  |  |  |  |  |
| Intercept | 4.2083 | 0.1277 | 32.952 | < 0.001 | s(O_var) | 1 | 2.713 | 0.09 |
| Gender (M) | 0.9066 | 0.1519 | 5.966 | < 0.001 | s(E_var) | 1 | 0.06 | > 0.05 |
| France | -0.1627 | 0.241 | -0.675 | > 0.05 | s(N_var) | 1 | 3.541 | 0.06 |
| UK & Ireland | 1.4801 | 0.1646 | 8.993 | < 0.001 | s(C_var) | 1.02 | 0.459 | > 0.05 |
|  |  |  |  |  | s(A_var) | 1 | 0.769 | > 0.05 |
|  |  |  |  |  | s(I_var) | 1 | 0.005 | > 0.05 |
|  |  |  |  |  | s(AS_var) | 1.486 | 1.927 | > 0.05 |
|  |  |  |  |  | s(SS_var) | 1 | 0.335 | > 0.05 |
|  |  |  |  |  | s(H_var) | 3.823 | 2.229 | 0.09 |
|  |  |  |  |  |  |  |  |  |
| Model 7 | ∆ Alcohol use frequency (Middle-adolescence to Eerly-adulthood) ~ s(O_var) + s(E_var) + s(N_var) + s(C_var) + s(A_var) + s(I_var) + s(AS_var) + s(SS_var) + s(H_var) + gender + country | | | | | | | |
|  |  |  |  |  |  |  |  |  |
| Intercept | 0.3594 | 0.07888 | 4.556 | < 0.001 | s(O_var) | 1.298 | 0.173 | > 0.05 |
| Gender (M) | 0.25283 | 0.09484 | 2.666 | < 0.01 | s(E_var) | 4.739 | 1.773 | 0.07 |
| France | 0.57953 | 0.14845 | 3.904 | < 0.001 | s(N_var) | 2.437 | 2.133 | > 0.05 |
| UK & Ireland | 1.06424 | 0.1025 | 10.383 | < 0.001 | s(C_var) | 1 | 1.793 | > 0.05 |
|  |  |  |  |  | s(A_var) | 1 | 1.033 | > 0.05 |
|  |  |  |  |  | s(I_var) | 1 | 3.562 | 0.06 |
|  |  |  |  |  | s(AS_var) | 1 | 1.377 | > 0.05 |
|  |  |  |  |  | s(SS_var) | 1.865 | 1.153 | > 0.05 |
|  |  |  |  |  | s(H_var) | 1 | 1.135 | > 0.05 |
|  |  |  |  |  |  |  |  |  |
| Model 8 | ∆ Alcohol use frequency (Late-adolescence to Early-adulthood) ~ s(O_var) + s(E_var) + s(N_var) + s(C_var) + s(A_var) + s(I_var) + s(AS_var) + s(SS_var) + s(H_var) + gender + country | | | | | | | |
|  |  |  |  |  |  |  |  |  |
| Intercept | 0.13089 | 0.04092 | 3.199 | 0.001 | s(O_var) | 1 | 0.024 | > 0.05 |
| Gender (M) | 0.01524 | 0.04913 | 0.31 | > 0.05 | s(E_var) | 1 | 0.12 | > 0.05 |
| France | 0.26004 | 0.07705 | 3.375 | < 0.001 | s(N_var) | 2.24 | 1.391 | > 0.05 |
| UK & Ireland | 0.02151 | 0.05302 | 0.406 | > 0.05 | s(C_var) | 6.766 | 3.417 | 0.001 |
|  |  |  |  |  | s(A_var) | 1 | 0.538 | > 0.05 |
|  |  |  |  |  | s(I_var) | 1 | 0.43 | > 0.05 |
|  |  |  |  |  | s(AS_var) | 1.772 | 1.915 | > 0.05 |
|  |  |  |  |  | s(SS_var) | 1 | 0.472 | > 0.05 |
|  |  |  |  |  | s(H_var) | 1 | 0.152 | > 0.05 |

Table 3. Summary of supplementary generalized additive mixture models. O, openness to experience. E, extraversion. N, neuroticism. C, conscientiousness. A, agreeableness. I, impulsivity. AS, anxiety sensitivity. SS, sensation seeking. H, hopelessness. var, variance score.

| **Personality trait** | **Gender** | *n* | **Variance Score 1** | | **Variance Score 2** | |
| --- | --- | --- | --- | --- | --- | --- |
|  |  |  | *M* | *SD* | *M* | *SD* |
|  |  |  |  |  |  |  |
| Openness | F | *527* | 0.10 | 0.09 | 0.09 | 0.10 |
|  | M | *441* | 0.12 | 0.11 | 0.10 | 0.11 |
|  |  |  |  |  |  |  |
| Extraversion | F | *527* | 0.10 | 0.10 | 0.09 | 0.11 |
|  | M | *441* | 0.12 | 0.13 | 0.10 | 0.12 |
|  |  |  |  |  |  |  |
| Neuroticism | F | *527* | 0.21 | 0.20 | 0.19 | 0.21 |
|  | M | *441* | 0.19 | 0.18 | 0.17 | 0.17 |
|  |  |  |  |  |  |  |
| Conscientiousness | F | *527* | 0.15 | 0.16 | 0.13 | 0.16 |
|  | M | *441* | 0.15 | 0.15 | 0.13 | 0.17 |
|  |  |  |  |  |  |  |
| Agreeableness | F | *527* | 0.11 | 0.11 | 0.09 | 0.11 |
|  | M | *441* | 0.11 | 0.10 | 0.10 | 0.11 |
|  |  |  |  |  |  |  |
| Impulsivity | F | *527* | 0.12 | 0.13 | 0.11 | 0.14 |
|  | M | *441* | 0.11 | 0.12 | 0.11 | 0.14 |
|  |  |  |  |  |  |  |
| Sensation seeking | F | *527* | 0.12 | 0.13 | 0.11 | 0.14 |
|  | M | *441* | 0.11 | 0.12 | 0.10 | 0.13 |
|  |  |  |  |  |  |  |
| Anxiety sensitivity | F | *527* | 0.14 | 0.15 | 0.13 | 0.17 |
|  | M | *441* | 0.12 | 0.14 | 0.12 | 0.17 |
|  |  |  |  |  |  |  |
| Hopelessess | F | *527* | 0.11 | 0.12 | 0.10 | 0.13 |
|  | M | *441* | 0.12 | 0.14 | 0.10 | 0.14 |

Table 4. Descriptive statistics of variance in personality scores across Early-adolescence through to Early-adulthood (Variance Score 1) and Early-adolescence through to Late-adulthood (Variance Score 2).

Table 5. Descriptive statistics of alcohol use and personality factors across pre-COVID and COVID timepoints

|  | **Sex** | *n* | **BL** | |  | **FU1** | |  | **FU2** | |  | **FU3** | | *n* | **COVID-T1** | | *n* | **COVID-T2** | |
| --- | --- | --- | --- | --- | --- | --- | --- | --- | --- | --- | --- | --- | --- | --- | --- | --- | --- | --- | --- |
|  |  |  | *M* | *SD* |  | *M* | *SD* |  | *M* | *SD* |  | *M* | *SD* |  | *M* | *SD* |  | *M* | *SD* |
| Age | F | *527* | 13.92 | 0.40 |  | 16.07 | 0.60 |  | 18.40 | 0.65 |  | 22.00 | 0.63 | *239* | 25.05 | 0.82 | *155* | 25.65 | 0.75 |
|  | M | *441* | 13.94 | 0.41 |  | 16.01 | 0.59 |  | 18.43 | 0.65 |  | 21.99 | 0.69 | *157* | 24.93 | 0.80 | *89* | 25.39 | 0.76 |
|  |  |  |  |  |  |  |  |  |  |  |  |  |  |  |  |  |  |  |  |
| Alcohol Use | F | *527* | 1.93 | 1.36 |  | 3.44 | 2.01 |  | 4.74 | 2.37 |  | 4.67 | 2.08 | *239* | 3.65 | 2.23 | *155* | 3.83 | 1.98 |
|  | M | *441* | 1.92 | 1.38 |  | 4.04 | 2.49 |  | 5.66 | 2.55 |  | 5.70 | 2.25 | *157* | 4.27 | 2.56 | *89* | 4.91 | 2.08 |
|  |  |  |  |  |  |  |  |  |  |  |  |  |  |  |  |  |  |  |  |
| Openness | F | *527* | 2.29 | 0.47 |  | 2.35 | 0.49 |  | 2.42 | 0.51 |  | 2.50 | 0.53 |  |  |  |  |  |  |
|  | M | *441* | 2.14 | 0.48 |  | 2.26 | 0.53 |  | 2.41 | 0.55 |  | 2.53 | 0.55 |  |  |  |  |  |  |
|  |  |  |  |  |  |  |  |  |  |  |  |  |  |  |  |  |  |  |  |
| Extraversion | F | *527* | 2.53 | 0.50 |  | 2.46 | 0.50 |  | 2.46 | 0.49 |  | 2.41 | 0.53 |  |  |  |  |  |  |
|  | M | *441* | 2.44 | 0.46 |  | 2.43 | 0.51 |  | 2.45 | 0.47 |  | 2.39 | 0.53 |  |  |  |  |  |  |
|  |  |  |  |  |  |  |  |  |  |  |  |  |  |  |  |  |  |  |  |
| Neuroticism | F | *527* | 2.07 | 0.61 |  | 2.06 | 0.66 |  | 1.93 | 0.70 |  | 1.93 | 0.74 |  |  |  |  |  |  |
|  | M | *441* | 1.73 | 0.58 |  | 1.65 | 0.61 |  | 1.50 | 0.62 |  | 1.52 | 0.69 |  |  |  |  |  |  |
|  |  |  |  |  |  |  |  |  |  |  |  |  |  |  |  |  |  |  |  |
| Conscientiousness | F | *527* | 2.36 | 0.57 |  | 2.42 | 0.60 |  | 2.59 | 0.61 |  | 2.75 | 0.58 |  |  |  |  |  |  |
|  | M | *441* | 2.32 | 0.57 |  | 2.35 | 0.59 |  | 2.44 | 0.59 |  | 2.57 | 0.60 |  |  |  |  |  |  |
|  |  |  |  |  |  |  |  |  |  |  |  |  |  |  |  |  |  |  |  |
| Agreeableness | F | *527* | 2.52 | 0.43 |  | 2.58 | 0.43 |  | 2.76 | 0.44 |  | 2.89 | 0.44 |  |  |  |  |  |  |
|  | M | *441* | 2.38 | 0.41 |  | 2.43 | 0.46 |  | 2.54 | 0.48 |  | 2.66 | 0.46 |  |  |  |  |  |  |
|  |  |  |  |  |  |  |  |  |  |  |  |  |  |  |  |  |  |  |  |
| Impulsivity | F | *527* | 2.40 | 0.44 |  | 2.24 | 0.43 |  | 2.17 | 0.42 |  | 2.05 | 0.43 |  |  |  |  |  |  |
|  | M | *441* | 2.37 | 0.41 |  | 2.25 | 0.43 |  | 2.21 | 0.43 |  | 2.13 | 0.45 |  |  |  |  |  |  |
|  |  |  |  |  |  |  |  |  |  |  |  |  |  |  |  |  |  |  |  |
| Sensation seeking | F | *527* | 2.59 | 0.50 |  | 2.62 | 0.53 |  | 2.62 | 0.53 |  | 2.62 | 0.58 |  |  |  |  |  |  |
|  | M | *441* | 2.76 | 0.48 |  | 2.77 | 0.48 |  | 2.84 | 0.51 |  | 2.85 | 0.53 |  |  |  |  |  |  |
|  |  |  |  |  |  |  |  |  |  |  |  |  |  |  |  |  |  |  |  |
| Anxiety sensitivity | F | *527* | 2.35 | 0.46 |  | 2.33 | 0.48 |  | 2.45 | 0.47 |  | 2.42 | 0.53 |  |  |  |  |  |  |
|  | M | *441* | 2.14 | 0.46 |  | 2.13 | 0.44 |  | 2.24 | 0.46 |  | 2.22 | 0.51 |  |  |  |  |  |  |
|  |  |  |  |  |  |  |  |  |  |  |  |  |  |  |  |  |  |  |  |
| Hopelessness | F | *527* | 1.88 | 0.40 |  | 1.88 | 0.45 |  | 1.91 | 0.47 |  | 1.84 | 0.51 |  |  |  |  |  |  |
|  | M | *441* | 1.84 | 0.37 |  | 1.84 | 0.44 |  | 1.83 | 0.47 |  | 1.82 | 0.53 |  |  |  |  |  |  |

|  | **Parametric coefficients** |  |  |  |  | **Smooth terms** | |  |
| --- | --- | --- | --- | --- | --- | --- | --- | --- |
|  | **Estimate** | **Std. Error** | **t-value** | **p-value** |  | **EDF** | **F-stat** | **p-value** |
| Model 1 | Alcohol use frequency (COVID-T1) ~ s(O_var) + s(E_var) + s(N_var) + s(C_var) + s(A_var) + s(I_var) + s(AS_var) + s(SS_var) + s(H_var) + sex + country | | | | | | | |
| Intercept | 3.22 | 0.22 | 14.49 | < 0.001 | s(O_var) | 1.54 | 0.91 | > 0.05 |
| Sex (M) | 0.50 | 0.25 | 2.05 | < 0.05 | s(E_var) | 1.00 | 0.63 | > 0.05 |
| France | 1.10 | 0.32 | 3.47 | < 0.001 | s(N_var) | 1.00 | 1.11 | > 0.05 |
| UK & Ireland | 0.52 | 0.28 | 1.85 | 0.06 | s(C_var) | 1.00 | 0.09 | > 0.05 |
|  |  |  |  |  | s(A_var) | 1.00 | 0.89 | > 0.05 |
|  |  |  |  |  | s(I_var) | 1.00 | 0.65 | > 0.05 |
|  |  |  |  |  | s(AS_var) | 1.30 | 2.35 | 0.08 |
|  |  |  |  |  | s(SS_var) | 1.00 | 0.26 | > 0.05 |
|  |  |  |  |  | s(H_var) | 1.96 | 1.24 | > 0.05 |
| Model 2 | Alcohol use frequency (COVID-T2) ~ s(O_var) + s(E_var) + s(N_var) + s(C_var) + s(A_var) + s(I_var) + s(AS_var) + s(SS_var) + s(H_var) + sex + country | | | | | | | |
| Intercept | 3.35 | 0.24 | 14.13 | < 0.001 | s(O_var) | 2.00 | 1.83 | > 0.05 |
| Sex (M) | 1.01 | 0.27 | 3.77 | < 0.001 | s(E_var) | 1.00 | 0.97 | > 0.05 |
| France | 0.55 | 0.31 | 1.78 | 0.07 | s(N_var) | 1.43 | 1.94 | 0.09 |
| UK & Ireland | 0.99 | 0.33 | 3.04 | < 0.01 | s(C_var) | 1.00 | 0.37 | > 0.05 |
|  |  |  |  |  | s(A_var) | 1.00 | 0.23 | > 0.05 |
|  |  |  |  |  | s(I_var) | 1.00 | 0.72 | > 0.05 |
|  |  |  |  |  | s(AS_var) | 1.86 | 2.76 | < 0.05* |
|  |  |  |  |  | s(SS_var) | 1.00 | 0.16 | > 0.05 |
|  |  |  |  |  | s(H_var) | 2.25 | 2.37 | 0.08 |
| Model 3 | ∆ Alcohol use frequency (FU3 to COVID-T1) ~ s(O_var) + s(E_var) + s(N_var) + s(C_var) + s(A_var) + s(I_var) + s(AS_var) + s(SS_var) + s(H_var) + sex + country | | | | | | | |
| Intercept | -0.17 | 0.05 | -3.71 | < 0.001 | s(O_var) | 1.00 | 0.10 | > 0.05 |
| sex (M) | -0.07 | 0.05 | -1.48 | > 0.05 | s(E_var) | 1.61 | 0.80 | > 0.05 |
| France | 0.17 | 0.06 | 2.64 | < 0.01 | s(N_var) | 1.00 | 0.04 | > 0.05 |
| UK & Ireland | -0.08 | 0.06 | -1.43 | > 0.05 | s(C_var) | 1.00 | 0.04 | > 0.05 |
|  |  |  |  |  | s(A_var) | 1.00 | 0.06 | > 0.05 |
|  |  |  |  |  | s(I_var) | 1.00 | 3.47 | 0.06 |
|  |  |  |  |  | s(AS_var) | 1.00 | 1.37 | > 0.05 |
|  |  |  |  |  | s(SS_var) | 1.00 | 0.50 | > 0.05 |
|  |  |  |  |  | s(H_var) | 1.00 | 0.06 | > 0.05 |
| Model 4 | ∆ Alcohol use frequency (FU3 to COVID-T2) ~ s(O_var) + s(E_var) + s(N_var) + s(C_var) + s(A_var) + s(I_var) + s(AS_var) + s(SS_var) + s(H_var) + sex + country | | | | | | | |
| Intercept | -0.20 | 0.06 | -3.43 | < 0.001 | s(O_var) | 1 | 0.00 | > 0.05 |
| Sex (M) | -0.02 | 0.06 | -0.35 | > 0.05 | s(E_var) | 1 | 0.05 | > 0.05 |
| France | 0.19 | 0.07 | 2.51 | < 0.05 | s(N_var) | 1 | 0.06 | > 0.05 |
| UK & Ireland | 0.07 | 0.08 | 0.91 | > 0.05 | s(C_var) | 1 | 0.99 | > 0.05 |
|  |  |  |  |  | s(A_var) | 1 | 0.10 | > 0.05 |
|  |  |  |  |  | s(I_var) | 1 | 1.06 | > 0.05 |
|  |  |  |  |  | s(AS_var) | 1 | 1.72 | > 0.05 |
|  |  |  |  |  | s(SS_var) | 1 | 0.03 | > 0.05 |
|  |  |  |  |  | s(H_var) | 1 | 1.57 | > 0.05 |
| Model 5 | Alcohol use frequency (FU3) ~ s(O_var) + s(E_var) + s(N_var) + s(C_var) + s(A_var) + s(I_var) + s(AS_var) + s(SS_var) + s(H_var) + sex + country | | | | | | | |
| Intercept | 4.17 | 0.11 | 36.97 | < 0.001 | s(O_var) | 1.00 | 9.86 | < 0.001** |
| Sex (M) | 0.96 | 0.14 | 7.08 | < 0.001 | s(E_var) | 1.27 | 0.06 | > 0.05 |
| France | 0.27 | 0.21 | 1.29 | > 0.05 | s(N_var) | 1.30 | 0.20 | > 0.05 |
| UK & Ireland | 1.33 | 0.15 | 9.10 | < 0.001 | s(C_var) | 1.60 | 0.50 | > 0.05 |
|  |  |  |  |  | s(A_var) | 2.04 | 0.93 | > 0.05 |
|  |  |  |  |  | s(I_var) | 1.00 | 1.96 | > 0.05 |
|  |  |  |  |  | s(AS_var) | 1.00 | 0.03 | > 0.05 |
|  |  |  |  |  | s(SS_var) | 1.00 | 0.80 | > 0.05 |
|  |  |  |  |  | s(H_var) | 1.79 | 0.36 | > 0.05 |
|  |  |  |  |  |  |  |  |  |

Table 6. Summary of generalized additive mixture models. O, openness to experience. E, extraversion. N, neuroticism. C, conscientiousness. A, agreeableness. I, impulsivity. AS, anxiety sensitivity. SS, sensation seeking. H, hopelessness.

| **Model** | **Outcome** | **Predictor(s)** | **Covariates** | **Interaction tested** |
| --- | --- | --- | --- | --- |
| Model 1 | Alcohol use (COVID-T1) | Personality variability (all traits) | Sex, country | Yes (sex × predictors) |
| Model 2 | Alcohol use (COVID-T2) | Personality variability | Sex, country | Yes |
| Model 3 | Δ Alcohol (FU3 → COVID-T1) | Personality variability | Sex, country | Yes |
| Model 4 | Δ Alcohol (FU3 → COVID-T2) | Personality variability | Sex, country | Yes |
| Model 5 | Alcohol use (FU3) | Personality variability | Sex, country | Yes |
| Model 6 | Alcohol use (late adolescence) | Personality variability | Sex, country | Yes |
| Model 7 | Δ Alcohol (mid → early adulthood) | Personality variability | Sex, country | Yes |
| Model 8 | Δ Alcohol (late → early adulthood) | Personality variability | Sex, country | Yes |
